# Non Destructive Characterization of Cortical Bone Micro-Damage by Nonlinear Resonant Ultrasound Spectroscopy

**DOI:** 10.1371/journal.pone.0083599

**Published:** 2014-01-02

**Authors:** Sylvain Haupert, Sandra Guérard, Françoise Peyrin, David Mitton, Pascal Laugier

**Affiliations:** 1 UPMC Univ Paris 06, CNRS UMR7623, Laboratoire d’Imagerie Paramétrique, Paris, France; 2 Arts et Métiers ParisTech, LBM, Paris, France; 3 CREATIS, INSERM U1044, CNRS 5220, INSA Lyon, Université Lyon 1, Lyon, France; 4 European Synchrotron Radiation Facility, Grenoble, France; 5 Université de Lyon, IFSTTAR, LBMC, UMR_T 9406, Université Lyon 1, Lyon, France; University of Notre Dame, United States of America

## Abstract

The objective of the study was to evaluate the ability of a nonlinear ultrasound technique, the so-called nonlinear resonant ultrasound spectroscopy (NRUS) technique, for detecting early microdamage accumulation in cortical bone induced by four-point bending fatigue. Small parallelepiped beam-shaped human cortical bone specimens were subjected to cyclic four-point bending fatigue in several steps. The specimens were prepared to control damage localization during four-point bending fatigue cycling and to unambiguously identify resonant modes for NRUS measurements. NRUS measurements were achieved to follow the evolution of the nonlinear hysteretic elastic behavior during fatigue-induced damage. After each fatigue step, a small number of specimens was removed from the protocol and set apart to quantitatively assess the microcrack number density and length using synchrotron radiation micro-computed tomography (SR-µCT). The results showed a significant effect of damage steps on the nonlinear hysteretic elastic behavior. No significant change in the overall length of microcracks was observed in damaged regions compared to the load-free control regions. Only an increased number of shortest microcracks, those in the lowest quartile, was noticed. This was suggestive of newly formed microcracks during the early phases of damage accumulation. The variation of nonlinear hysteretic elastic behavior was significantly correlated to the variation of the density of short microcracks. Our results suggest that the nonlinear hysteretic elastic behavior is sensitive to early bone microdamage. Therefore NRUS technique can be used to monitor fatigue microdamage progression in *in vitro* experiments.

## Introduction

Among various bone quality factor, bone microdamage is certainly the less understood, as *in vivo* microcracks detection is still challenging. Bone microdamage is a natural phenomenon caused by daily loading [1]. Microdamage manifests as linear microcracks and diffuse damage in cortical bone [2], [3]. The density of linear microcrack increases significantly with age in cortical bone [2], [4]–[6]. While microdamage is of little consequence under normal bone self-repair capability [7], microdamage accumulation following impaired repair capabilities caused by disease, age or drug absorption [8]–[10] is suspected to reduce bone biomechanical competence, including toughness [11], [12], stiffness [11]–[16] and ultimate load [11], [17]. Such alterations may lead ultimately to an increase in fracture risk [6]. Histomorphometry is the current gold standard to characterize microdamage *in vitro* [2], [14]. However, quantitative assessment of microcracks with histomorphometry entails serial sectioning and observer intervention, which is usually time consuming. Moreover, as microcracks are relatively scarce in bone 2-D cross-sections, the statistical validity remains challenging [18].

Recently, synchrotron radiation micro-computed tomography (SR-µCT) enabled the 3-D assessment of microcracks at a micro-scale resolution [19]–[22]. These techniques are inherently destructive and cannot be used to study microdamage *in vivo*.

Several measurement modalities, including positron emission tomography (PET) [23], [24], nuclear magnetic resonance NMR T2 relaxation time [25] and nonlinear ultrasound [26], [27] are currently explored to non destructively assess microdamage in living bones.

Quantitative ultrasound is widely used to assess skeletal status [28]. However, the linear elastic (speed of sound) and dissipative (attenuation) parameters derived in quantitative ultrasound are not sensitive to damage [29]–[32]. Contrary to linear acoustics, in the framework of nonlinear acoustics, the propagation velocity and the attenuation (or dissipation) of acoustic waves are amplitude dependent. Those peculiarities give rise to various phenomena called nonlinear acoustical effects. Damaged materials have proved to exhibit a characteristic nonlinear behavior that can be used to infer material mechanical integrity. Elastic nonlinear parameters derived from dynamic wave studies were found to be far more sensitive than their linear counterparts to damage in a variety of materials [33], [34]. This has recently motivated several research groups to adapt ultrasound-based nonlinear dynamic elastic testing methods to assess the level of microdamage in cortical or cancellous bone using different techniques. These include nonlinear ultrasonic resonant spectroscopy (NRUS) [35]–[37], dynamic acousto-elasticity testing (DAET) [38]–, harmonic generation [26], [27] and nonlinear wave modulation spectroscopy [41], [42]. The advantage of these nonlinear techniques is that they are inherently non destructive and can therefore potentially be implemented *in vivo* [26], [27].

In previous NRUS studies, our group has reported that, under resonance conditions, the resonance frequency of damaged femoral diaphysis was down shifted with increasing vibration amplitude. We found a correlation of progressive fatigue of human bone samples to their nonlinear dynamical response [35]–[37]. Such an effect can be interpreted as a softening of the material in presence of cracks when the wave amplitude increases gradually (i.e., the modulus of the material decreases with dynamical forcing). This softening effect rises as the elastic non linearity (i.e., the level of damage) of the material increases [43]. With the possibility to non-invasively evaluate nonlinear properties assumed to be related to microdamage accumulation, NRUS is an attractive technique to evaluate microdamage in bone. In the above mentioned NRUS studies, nonlinear ultrasonic measurements were not validated by histology nor by high resolution µCT. The measured nonlinear elastic properties could not be correlated to microdamage characteristics. Therefore the goal of this study was to assess the relationships between the nonlinear elastic parameter and microdamage characteristics on human cortical bone specimens subjected to fatigue loading with a specific focus on assessment of the sensitivity of the technique to early phase of damage accumulation.

## Materials and Methods

### Specimen preparation and measuring protocol

Small parallelepiped beam-shaped human cortical bone specimens were subjected to cyclic four-point bending fatigue in several steps. The specimens were prepared to control damage localization during four-point bending fatigue cycling and to unambiguously identify resonant modes for NRUS measurements.

Fourteen human cortical bone specimens were prepared from the femoral mid-diaphysis of four female donors (78, 80, 98, 98 years old). Ethical approval for the collection of samples was granted by the Human Ethics Committee of the Centre du don des Corps at the University Paris Descartes (Paris, France). The tissue donors or their legal guardians provided informed written consent to give their tissue for investigation, in accord with legal clauses stated in the French Code of Public Health. The specimens were wet machined (Isomet 4000, Buehler GmbH, Düsseldorf, Germany) as parallelepiped beams (50*4*2 mm), defatted [44] and stored at −20°C until experiments.

Apparent dry density (*ρ_dry_*) was evaluated by measuring the specimen volume and weight. Bone specimens were dried at 37°C during one night in a climate chamber (Memmert GmbH HCP 108, Schwabach, Germany) at relative humidity 15% in the presence of desiccators. Drying and rewetting procedure does not affect bone properties as the collagen molecular structure remains intact [45], [46].

**Figure 1:**
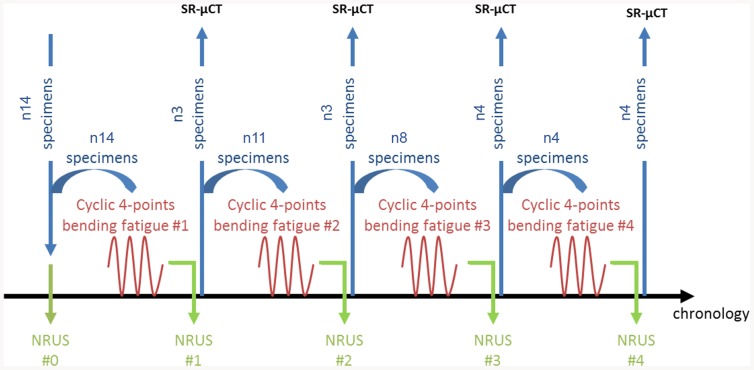
Diagram illustrating the experimental protocol.

The procedure for the NRUS and mechanical studies began with the initial NRUS measurements for all specimens to determine the initial nonlinearity of the material. The specimens were then taken through a damage step, consisting of cyclic four-point bending as described below, during which mechanical parameters were determined. NRUS measurements were repeated after each cycling session. Four damage steps were achieved. After each damage step, three or four specimens were removed for future 3-D SR-µCT investigations of microdamage characteristics. The measurement protocol is illustrated in Fig. 1.

### NRUS measurements

**Figure 2:**
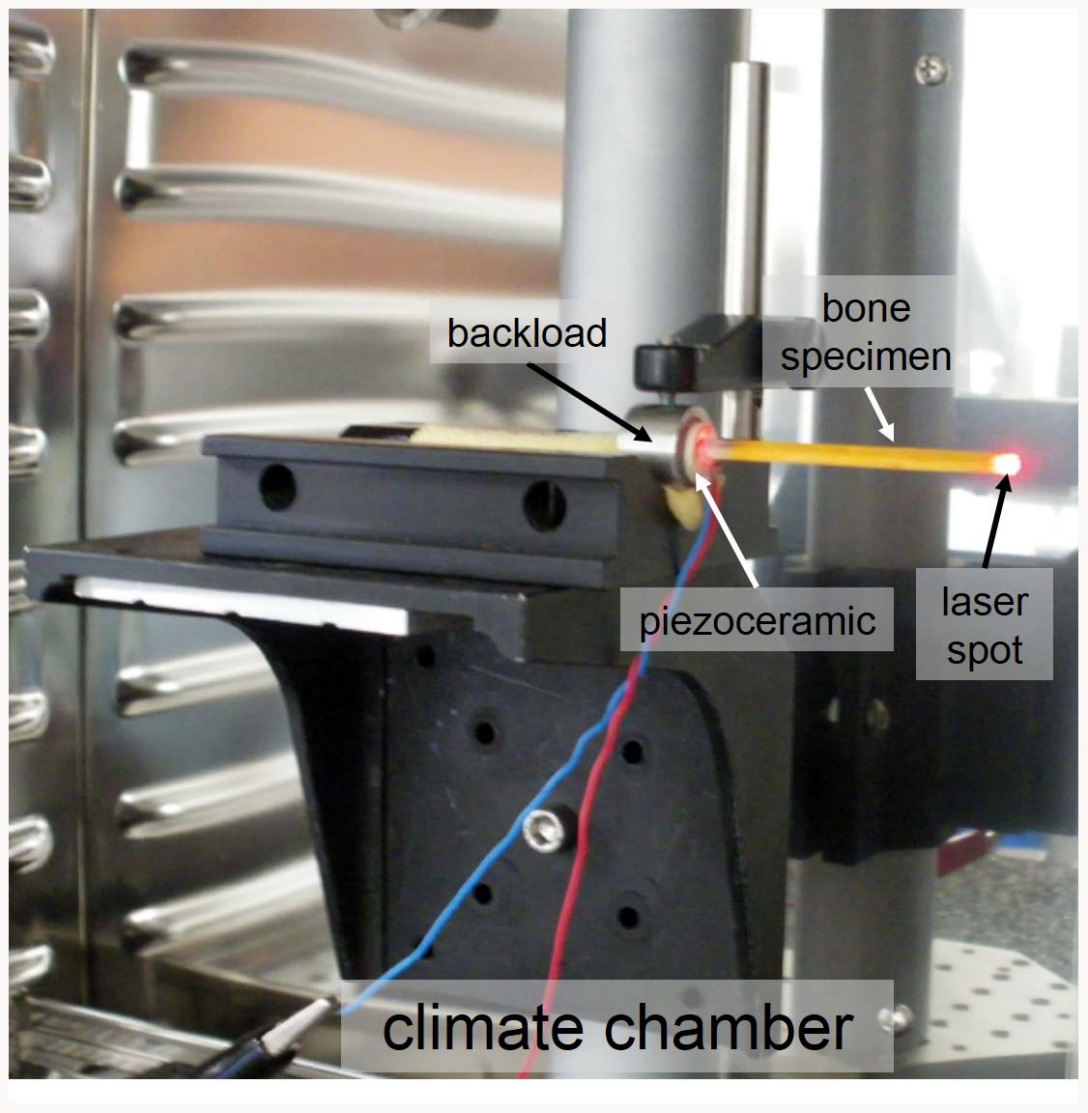
NRUS experimental setup. Bone specimen bonded on a piezoceramic emitter is placed in a climate chamber.



The principles of NRUS measurements have been extensively described elsewhere [43]. Briefly, a piezoceramic emitter (Fuji Ceramics Corporation, Yamamiya, Japan) glued on a backload (i.e. a heavy mass compared to the specimen) was bonded at one end of the specimens to ensure free-fix boundary conditions for NRUS measurements (Fig. 2). Each specimen was excited by a swept-sine (M2i.6012, Spectrum GmbH, Grosshansdorf, Germany) encompassing the first resonant modes of the cortical beam (assumed to be pure compression modes under asymmetric loading conditions). The dynamic strain amplitude *ε* was calculated from the longitudinal particle displacement *U* at one end of the sample measured by a laser vibrometer (LSV 1MHz, SIOS, Ilmenau, Germany):

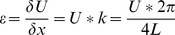
(1)where *k* is the wave number and *L* is the specimen length. The resonance peak frequency *f* of the first compression mode was derived for each voltage drive level from the strain amplitude measured as a function of frequency at the corresponding excitation level. From the resonance peak data, the nonlinear parameter *α_f_* can be calculated using the following equation [43], [47]:
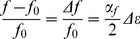
(2)where *f* is the resonance frequency at increased strain level and *f_0_* its corresponding value at the lowest drive amplitude [47]. Eq. 2 expresses that the frequency shift Δ*f* is proportional to the peak strain amplitude Δ*ε* via the nonlinear elastic *α_f_* parameter. This parameter, so-called nonlinear hysteretic elastic parameter, is typical of nonlinearities that appear for strains above approximately 10^−5^ [47] in damaged materials. It conveys information about the amount of hysteretic nonlinearity directly linked to damage accumulation in a material.

The widely used NRUS measurement protocol [43] was optimized to achieve high sensitivity. The measurement of the reference resonance frequency *f_0_* used to compute *α_f_* was repeated before each excitation level. In doing so, the measurements become less affected by changes in environmental conditions such as temperature and yields more precise and stable *α_f_* estimates [48].

During the NRUS measurements, specimens were kept at fixed temperature (37°C ±0.1°C) and relative humidity (15%±5%) into a climate chamber (Fig. 2)

### Biomechanical testing

The piezoceramic emitter attached to the specimen for NRUS measurements was removed and the specimen was rehydrated during 48 hours before each mechanical testing. All specimens were progressively damaged by cyclic four-point bending at 2 Hz under load control in a saline solution at 37°C (±1°C) using a hydraulic testing machine (INSTRON, 8802, High Wycombe, England) with a 1 kN loading cell (accuracy 0.5%) and the internal displacement transducer (accuracy 1%). The specific four-point bending assembly composed of 6.35 mm diameter roller-bearings with a 40 mm outer span and a pivoting 20 mm inner span minimizes the formation of grooves under the rollers [49], [50]. In this configuration, damage is expected to occur specifically in the mid region of the sample [12], while the distal regions remaining intact may be used as control. Initial Young’s modulus was determined during pre-cycling after 20 cycles by measuring the slope of the linear portion of the last load-displacement curve.

From the initial Young’s modulus, the load (*Fmax*) corresponding to 5000µε (i.e. an initial strain rate of 20000µε/s) at the mid-span was computed for all specimens [12]. The four-point bending fatigue was then applied between -10N and –*Fmax*. During the cycling session, the load and displacement curves were recorded to extract the linear elastic beam theory (LEBT) modulus (*E_LEBT_*) as defined by Landrigan [51]. *E_LEBT_* is a combination of elastic (secant modulus) and plastic (residual strain) biomechanical parameters. After each damage step, the *E_LEBT_* modulus is normalized by the initial value measured for the first loading cycle of the first damage step. *E_LEBT_* has been shown to decrease as bone microdamage accumulates [15], [16], [53]. The progressive damage was performed in four steps (one step = one cycling session), each step was defined by multi-criteria: 10% decrease of the *E_LEBT_* or pre-determined number of cycles ( = 6000) or anomalous *E_LEBT_* decreasing speed. This multi-criteria definition of each step was chosen to achieve progressive damage accumulation and to avoid specimen failure before the end of the fourth step.

### 3-D synchrotron radiation µCT (SR-µCT)

At the end of each step, a subset of 3 or 4 bone specimens was measured by SR-µCT at the European Synchrotron Radiation Facility in Grenoble, France. Two different reconstructed volumes of interest (VOI) were investigated. VOI1, located in the load-free region at one distal end of the sample, outside of the roller-support, was assumed to be free of damage (except for pre-existing initial pre-fatigue damage). VOI2, located in the central portion of the beam, is the region where most microdamage was assumed to accumulate during mechanical fatigue. The photon energy was 25 KeV and the size of the VOI was 2.8×2.8×1.96mm^3^ with a voxel size of 1.4 µm^3^. A set of 2500 projections were acquired at an angular step of 0.144°. The 2048×2048×1400 3D images were reconstructed using a filtered back projection algorithm and the contrast was linearly rescaled to an 8-bits dynamic to save memory storage. For microdamage characterization, the size of the investigated volume of both VOIs was reduced to 2.2×2.0×1.96 mm^3^.

**Figure 3:**
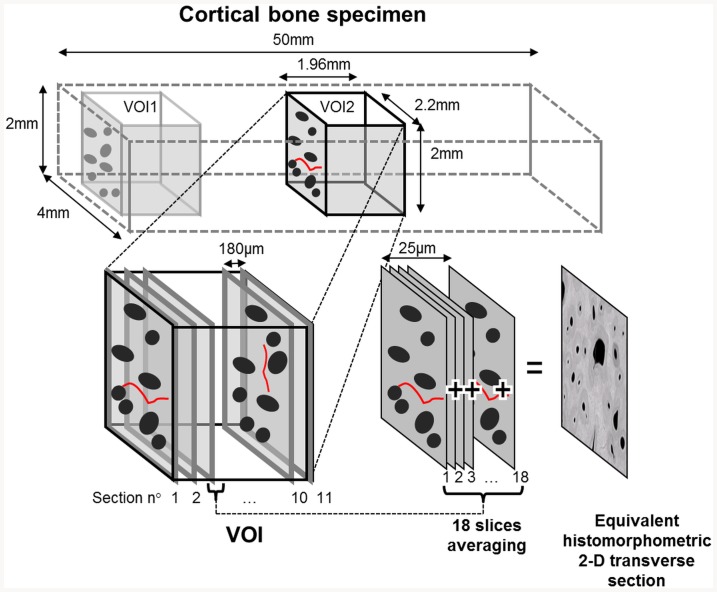
Diagram illustrating the process leading to an equivalent histomorphometric 2-D transverse cross-section image from 3-D reconstructed bone volumes acquired by SR-µCT.

Microdamage is generally characterized on transverse or longitudinal 2-D sections by conventional or epifluorescence microscopy [12], [14]. In this study, microdamage characteristics were quantitatively assessed in cortical bone volumes reconstructed from SR-µCT data. To this end, each VOI was sampled by eleven 2-D transverse cross-sections regularly spaced with an interval of 180µm (Fig. 3). Each cross-section was obtained by averaging a stack of 18 adjacent 1.4µm-thick slices in order to achieve transverse cross-section images with a depth of field of 25 µm equivalent to that achieved with epifluorescence microscopy. The averaging process has also the advantage to decrease the noise level and to improve to contrast between the microcracks and the bone matrix.

The surface microcrack density (Cr.Dn [#.mm^2^]) and microcrack length (Cr.Le [µm]) were measured using the software ImageJ (NIH, USA) with the plugin NeuronJ [52]. The bone surface was computed as the total area of bone section, including the pores (Haversian and Volkmann canals, and resorption cavities) as it is reported in the literature [8], [54]. The pores appeared as dark pixels and were clearly evidenced as one peak of the bimodal gray level histograms of the image. Thus they could easily be separated from bone tissue using a thresholding method to keep only low grey level pixels, with a threshold set to 100 (arbitrary unit) in the range [0–255] according to the histogram. The porosity value corresponds to the number of segmented pixels (pores) over the total surface of cortical bone including pores (in pixels).

Microcracks characteristics were determined first by including all microcracks. In a second step, microcracks fully embedded within the bone volume were processed separately from microcracks leading to the surface as they are mainly artifactual microcracks formed during the preparation process [14], [53], [55].

### Data analysis

Matlab 7.8 with statistics toolbox 7.6 (Mathworks, Natick, MA, USA) was used for statistical analyses. A non-parametric one-way analysis of variance (ANOVA) dedicated to repeated measurements (Friedman Test) followed by post hoc multiple comparison (Nemenyi test) was applied to test whether the levels of nonlinear elasticity achieved at each steps of the fatigue protocol were statistically significantly different. This analysis was performed on two subsets of the total set of specimens (N = 14). Group 1 (N = 8) includes all specimens having undergone the first three damage steps and Group 2 (N =  4) includes all specimens having undergone all the four damage steps. The effect of fatigue loading on microdamage characteristics (Cr.Dn and Cr.Le) in the control region and the damaged region was investigated with a non-parametric Wilcoxon signed rank test for all specimens (N = 14). The relationship of the nonlinear elastic parameter *α_f_* with microdamage characteristics was obtained by a Spearman correlation test taking into account all specimens (N = 14) as well as after removing the outlier (N = 13). P-values less than 0.05 were considered significant.

## Results

Individual results for mechanical and NRUS testing are presented in Table 1. In Table 2, we report only the microdamage characteristics for fully embedded microcracks as the results were similar when all microcracks were included in the analysis or after excluding those leading to the specimen surface.

**Table 1:** Characteristics of the human cortical bone specimens: density (*ρ_dry_*_)_, initial mechanical modulus LEBT (*E_LEBT_*), number of damage steps, number of cycles, initial, intermediate and final nonlinear elastic parameters (*α_f_*).

	*Architectural*		*Mechanical (wet)*			*Nonlinear ultrasonic parameters (dry)*				
*Specimens*	*ρ_dry_* [g/cm3]	Porosity [%]	initial *E_LEBT_* [KPa]	final *E_LEBT_* [KPa]	*Total num of cycles*	*α_f_* initial	*α_f_* step 1	*α_f_* step 2	*α_f_* step 3	*α_f_* step 4
#1	1753	11.4	15215	nm	155	−6	−6.4			
#2	1897	13.2	17069	nm	120	−4.8	−4.7			
#3	1870	6.3	17294	16027	906	−15.5	-15.3			
#4	1865	10.3	17382	12583	2100	−4.6	−5.9	−5.8		
#5	1353	26.5	9344	nm	175	−4.7	−6.4	-12.8		
#6	1854	12.4	15654	9799	1991	−4.4	−9.8	-15.4		
#7	1887	7.3	15722	11619	1788	−6	−9.7	-12.8	-16.2	
#8	2005	5.6	21857	14760	6226	−6.2	−6	−6.1	−6.5	
#9	1904	9.8	16598	14133	9220	−4.7	−5.7	−6.4	−7.1	
#10	1893	9.4	16296	11980	3275	−4.5	−4.6	−4.2	−6.2	
#11	1864	14.2	16055	9289	4221	−5	−4.4	−5	−5.9	−6
#12	1714	13.1	13470	7901	13826	−5.1	−6	−6.1	−6.5	−6.3
#13	1684	12.6	11876	7054	3616	−4.3	−4.9	−6.2	−6.1	−7.3
#14	1821	16.3	14406	11177	14396	−4.8	−5.2	−5.9	−6.6	-10.7
**Mean**	**1812**	**12**	**15588**	**11484**	**4430**	−**5.8**	−**6.8**	−**7.9**	−**6.4**	−**7.7**
**Std Dev**	**156**	**5.2**	**2885**	**4274**	**4816**	**2.9**	**3**	**3.8**	**2.3**	**2.2**
**Median**	**1864**	**11.9**	**15888**	**11619**	**2687**	−**4.8**	−**5.9**	−**6.1**	−**6.5**	−**6.8**

[nm = not measurable]

**Table 2:** Characteristics of microcracks embedded within the bone matrix in the control (VOI1) and fatigue-loaded (VOI2) volumes.

	Cr.Dn [#/mm^2^]		Cr.Le [µm]		Cr.Dn.Q1 [#/mm^2^]		Cr.Le.Q1 [µm]	
*Specimens*	VOI1	VOI2	VOI1	VOI2	VOI1	VOI2	VOI1	VOI2
#1	3.88	3.03	80.4	68.1	0.73	0.98	35.8	34.8
#2	3.16	2.76	64.1	65.4	0.72	0.6	27.8	34
#3	1.53	1.58	58.4	56.2	0.19	0.41	23.3	25.6
#4	1.97	1.77	69.7	69	0.35	0.53	31.5	31.8
#5	0.9	1.84	76.8	62.9	0.19	0.88	45.2	34.2
#6	3.17	2.84	85.9	70.6	0.67	0.91	38	36.3
#7	2.13	4.14	78.5	57.1	0.38	1.96	41.4	32.6
#8	0.36	0.44	99.5	72.5	0.08	0.18	46.7	42.5
#9	3.23	3.11	77.2	72.9	0.65	0.74	32.2	30.8
#10	2.6	4.59	73.1	95.5	0.43	0.45	31.3	31.6
#11	2.49	2.01	55.4	60.2	0.61	0.41	26.2	27.6
#12	0.23	0.25	51.1	54.4	0.02	0	17.3	-
#13	1.06	1.9	80.4	53.5	0.12	0.33	28.8	24.4
#14	1.08	1.99	66	61.6	0.16	0.61	30.3	27.9
**Mean**	**1.98**	**2.3**	**72.6**	**65.7**	**0.38**	**0.64**	**32.6**	**31.8**
**Std Dev**	**1.15**	**1.22**	**13**	**10.8**	**0.26**	**0.47**	**8.2**	**4.9**
**Median**	**2.05**	**2**	**74.9**	**64.1**	**0.36**	**0.56**	**31.4**	**31.8**

Cr.Dn and Cr.Le correspond to the microcracks density and their average length respectively. Cr.Dn.Q1 and Cr.Le.Q1 corresponds to the microcracks density and the average length of short microcracks, i.e. with length in the first quartile of each sample.

### Biomechanical testing

The variability of *E_LEBT_* has been assessed on five dedicated specimens that were not included in the protocol. They went through 20 cycles (after system stabilization) of four-point bending test. The process was repeated 6 times with repositioning. The coefficient of variation (standard deviation/mean) was found to be 6.3% for *E_LEBT_*.

The mechanical characteristics (Table 1) were found to vary between the specimens. The average initial *E_LEBT_* modulus was 15.1±3.0 GPa the average apparent dry density *ρ_dry_* was 1792±155 g.mm^−3^ and the average porosity was 12.0±5.2.

No significant trends could be observed between stopping criteria and the other measured variables (nonlinearity or microcracks characteristics).

### Ultrasonic (NRUS) measurements

The measurement precision of NRUS, assessed by the coefficient of variation of three measurements with intermediate debonding of the piezo-electric source and repositioning, was found to be 8.5% for *α_f_*. The initial nonlinear values of *α_f_* ranged between −4.3 and −6.2, except for one highly nonlinear specimen (*α_f_*  = −15.5). The average initial *α_f_* value was −5.8±2.9. After the last damage step (step 1 to 4 depending on the specimen), *α_f_* values ranged between −4.7 and −16.2.

On average, the nonlinear parameter *α_f_* increased with the number of fatigue steps. However, a disparity could be observed between the specimens.

**Figure 4:**
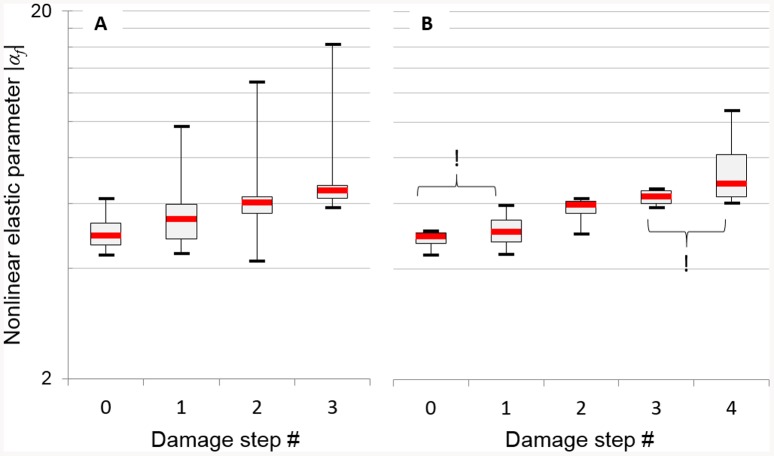
Box plot of the nonlinear elastic parameter *α_f_* after each damage step. (A) Group 1 (N = 8) specimens having undergone the first three stages of damage; (B) Group 2 (N = 4) specimens having undergone the four damage steps. (!) means no significant effect of fatigue on parameter *α_f_* between two steps (p>0.05).

A significant effect of fatigue on *α_f_* was measured for both groups (Group 1: p<0.05, no F value due to the number of steps less than four; Group 2, p = 0.01, F = 12.6). Group 1 including the eight specimens having undergone the first three stages of damage is represented in Fig. 4A. Group 2 including the four specimens having undergone all the four damage steps is represented in Fig. 4B. The result of the post-hoc comparison evidenced statistically significant variations of *α_f_* between damage steps for both groups except between the initial and first step and between the third and last step for specimens of the Group 2.

Finally, note that no correlation was found between the initial nonlinear elastic parameter *α_f_* or its variation Δ*α_f_*/*α_f_* and the initial elastic modulus *E_LEBT_*, the total number of cycles, the apparent density *ρ_dry_* nor the sample porosity.

### Microtomography

The total number of microcracks found by pooling the data of all the specimens and both VOIs was 4106 with 1380 microcracks leading to the specimen surface and 2726 microcracks fully embedded within the bone volume.

**Figure 5:**
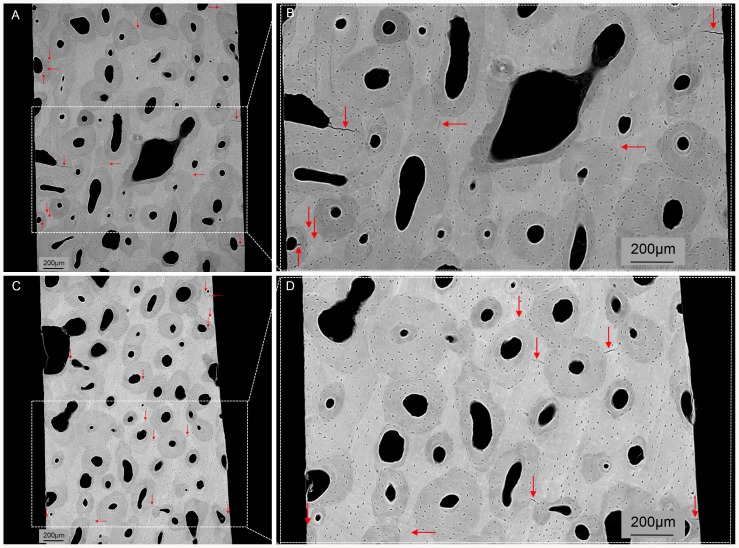
Example of a 2-D transverse cross-section extracted from the (A-B) unloaded region (VOI1) and (C-D) loaded region (VOI2) of the specimen #13. Red arrows point microcracks. Figures B and D are a zoom of the figures A and C respectively.

The difference in microdamage characteristics between VOI1 (control region) (Fig. 5A-B) and VOI2 (damage region) (Fig. 5C-D) did not reach statistical significance when all microcracks were included (VOI1: Cr.Le = 71.7±24.0µm; Cr.Dn = 3.11±1.51 #/mm^2^ and VOI2: Cr.Le = 69.6±25.7µm; Cr.Dn = 3.33±1.58#/mm^2^) nor after excluding from the analysis, microcracks leading to the specimen surface (VOI1: Cr.Le = 72.6±13.0µm; Cr.Dn = 1.98±1.15#/mm^2^ and VOI2: Cr.Le = 65.7±10.8µm; Cr.Dn = 2.30±1.22#/mm^2^). Damage characteristics of microcracks fully embedded in bone are summarized in Table 2. Note the large inter-specimen variability of Cr.Dn ranging between 0.23#/mm^2^ to 3.88#/mm^2^ (VOI1) and between 0.25#/mm^2^ to 4.59#/mm^2^ (VOI2).

**Figure 6:**
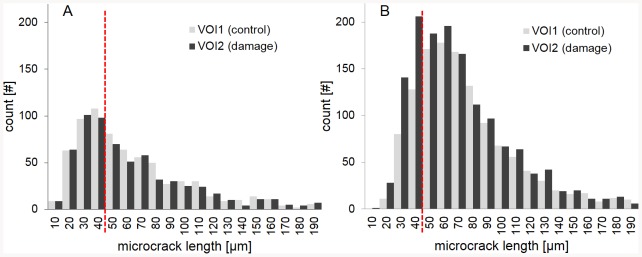
Distribution of microcracks length, in the control zone (VOI1) and the damage zone (VOI2) for all the fourteen specimens. (A) Only microcracks leading to the surface specimen are taken into account; (B) only microcracks fully embedded within the bone matrix are taken into account. In case of microcracks fully embedded within the bone matrix, there is a significant difference (p = 0.01) in the number of microcracks having a length shorter than 40µm between VOI1 and VOI2 in the damage zone.

In contrast, from the examination of the length distribution of microcracks, it appeared that fatigue cycling resulted in an increase of the density of the shortest microcracks, i.e., those in the first quartile (Q1), whereas the quantity of longer microcracks remained unchanged. The density Cr.Dn.Q1 of the shortest microcracks was significantly different between VOI1 and VOI2, both when all microcracks were included (VOI1: Cr.Dn.Q1 = 0.77±0.38#/mm^2^ and VOI2: Cr.Dn.Q1 = 1.06±0.60#/mm^2^; p<0.05) or when microcracks leading to the surface were excluded (VOI1: Cr.Dn.Q1 = 0.38±0.26#/mm^2^ and VOI2: Cr.Dn.Q1 = 0.64±0.47#/mm^2^; p<0.01) (Fig. 6B). The mean length Cr.Le.Q1 corresponding to the upper limit of the first quartile was found to be 32.6±8.2µm. Such a trend for short microcracks could not be observed for microcracks leading to the specimen surface (Fig. 6A).

**Figure 7:**
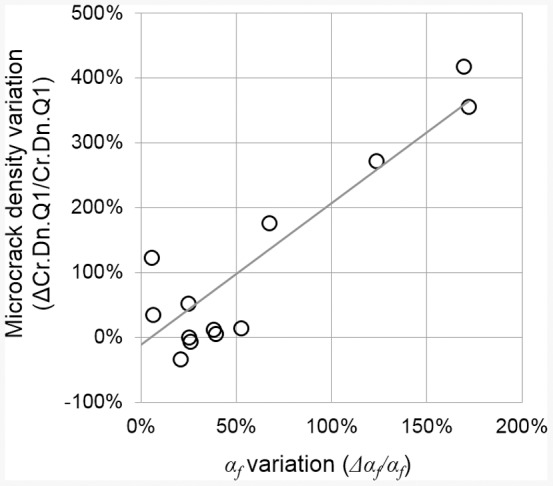
Correlation between the relative variation Δ*α_f_*/*α_f_* of the nonlinear elastic parameter (*α_f_* represents the difference between the initial value and the value measured after the last damage step) and the relative variation of short microcracks density ΔCr.Dn.Q1/Cr.Dn.Q1 between VOI1 and VOI2. One specimen (#6; ΔCr.Dn.Q1/Cr.Dn.Q1 = 0.36; Δ*α_f_*/*α_f_* = 2.48) exhibiting the strongest *α_f_* variation is not represented

The relative variation between VOI2 and VOI1 of density of short microcracks embedded within the bone matrix ΔCr.Dn.Q1/Cr.Dn.Q1 is plotted against the relative variation of the nonlinear parameter Δ*α_f_/α_f_* in Fig. 7. No significant correlation was found taken into account all the specimen (N = 14). However, when the outlier (specimen #6 exhibiting the strongest *α_f_* variation) was excluded, a significant correlation of r = 0.6 (*p*<0.05) was found between both quantities.

## Discussion

This is the first study reporting the nonlinear elastic hysteretic behavior (assessed by the nonlinear elastic parameter *α_f_*) and microdamage characteristics derived from SR-µCT, concurrently assessed in calibrated human cortical bone specimens during a four point-bending fatigue cycling protocol. By repeating the NRUS measurement protocol after each damage step, we were able to monitor the evolution of the nonlinear behavior during progressively induced mechanical damage, each specimen being its own control. We observed that the damage steps had a statistically significant effect on the nonlinear hysteretic parameter *α_f_* measured by NRUS. This suggests that the parameter *α_f_* is a sensitive marker to bone microdamage induced by *ex vivo* mechanical fatigue test. These results confirm the seminal observations made in previous studies by our group [35]–[37]. The present study brings a new insight by unraveling the link existing between the nonlinear elastic behavior and some damage characteristics, the latter being derived from µCT volumetric imaging. Our result evidenced that the increase of elastic non linearity was related to an increase of the density of the shortest microcracks embedded within the bone matrix, i.e., those in the first quartile.

### Origin of the elastic nonlinearity in bone

The nonlinear hysteretic elasticity (*α_f_*) of human cortical bone samples was measured in this study, allowing the comparison with other materials. Here, the nonlinear hysteretic elasticity typical of damaged materials is measured, as opposed to the classical elastic non linear response which exists in most materials, including undamaged solids, due to intrinsic anharmonicity of, for instance, the crystalline lattice-atomic level vibration.

The initial (pre-fatigue) nonlinear value (*α_f_* = −5.8±2.9) is consistent with the value previously reported for undamaged cortical bovine bone (*α_f_* = −5.0±2.5) [48]. This value is weak (ten times lower than that of intact polycrystal metals [48] or hundred times lower than for rocks [47] but not null, meaning that human cortical bone specimens exhibit low hysteretic elasticity behavior in pre-fatigue (native) configuration. Even after the progressive damage, the nonlinear behavior remains low when compared to other materials.

There are multiple sources of hysteretic nonlinearity in materials. Hysteresis in the dynamic strain-stress relationship is known to be produced by micro-friction, micro-adhesion and clapping due to presence of soft micro-structural features at different scales such as microcracks [56] or dislocations [57]. Such process could be at the origin of the observed pre- and post-damage bone nonlinearities. The sources are not known and were beyond the scope of this study. As bone is a hierarchical material [58], the process could take place at different level of the hierarchical structure:

1. at the nano-scale level, debonding of collagen fibers [59] could generate nonlinear effect as it is well known in fiber composite materials [34]. Stick-slip friction between collagen fibrils and nanocrystals [60]–[63] could also be a source of a hysteretic elastic behavior;
2. at the micro-scale level, nonlinear behavior could have its origin in the cement line sliding or osteon pull-out [64];
3. at the meso-scale level, micro and macrocracks might be the main structure generating nonlinear acoustic phenomena.

This list is not exhaustive but opens up about the multiplicity of factors behind the nonlinear hysteretic elastic behavior of cortical bone. As it stands, we cannot draw any conclusion about the origin of the native (here, “native” means “prior to any damage step”) nonlinear behavior of cortical bone. It may have its origin in damage, microcrack or diffuse type, native or induced by sample preparation. Such damage is observed by measuring the control regions located at the ends of the specimens. As for other potential sources of damage (dislocation, delamination, slip osteons, etc.), it would be necessary to assess their magnitude and explore them by dedicated experiments. However, as each sample is its own control, we can attribute unambiguously the increase in the nonlinear hysteretic behavior after fatigue to damage accumulation.

Besides, water saturation, capillarity effects and fluid flow pressure may also play a role as in rocks [65] and concrete [66] by modifying bone nonlinear elasticity, viscoelasticity and relaxation properties. For this reason, a particular attention was given to keep the samples at the same relative humidity (e.g. 15%±5%) during each NRUS experiment.

### Pre-existing microdamage (VOI1)

The density of microcracks (Cr.Dn) embedded within undamaged bone volumes (1.98±1.15#/mm^2^ in VOI1) is one order of magnitude larger compared to the values found in most studies on human cortical femur (0.21±0.16#/mm^2^ [5], 0.21±0.21#/mm^2^ [15], 0.15±0.16#/mm^2^ [67], 0.1±0.06#/mm^2^ [68]). Only two studies have shown comparable Cr.Dn ranging between 1#/mm^2^ to 5#/mm^2^ for women older than 45 years old and higher than 1.5#/mm^2^ for human femur older than 70 years old [4], [6]. However, one has to remind that microcracks density increases with age [2], [4]–[6], [67] and that the age of the donors in our study (88.5±9.8) is generally higher than in the above mention studies. This being said, even if we discarded microcracks leading to the surface, we cannot exclude that crack density can be artificially augmented by some artifactual microcracks formed during the preparation process of the specimens, especially those located 500µm from cutting edge [14], [53].

Factors other than age of donors and artifactual microcracks, however, can explain the difference between studies. Indeed, microcrack density may depend on the technique used to assess microdamage characteristics [69], [70]. For example, the number of microcracks counted by epifluorescence microscopy [70], [71] or by backscatter scanning electron (BSE) microscopy [69] could be up to twice the number measured by conventional microscopy based on basic Fuchsin dye. In this study, cortical bone microdamage quantitative assessment was done by SR-µCT. The contrast, resolution and depth of field of SR-µCT images differ from those of conventional optical microscopy-based histomorphometry approaches, which may affect differently the detection of microcracks and their characteristics. A face-to-face comparison between the different techniques would be warranted to provide an answer to this issue.

### Induced microdamage (VOI2)

When we considered all the detected microcracks, leading to the surface of the specimens and embedded within the bone matrix, we found no significant change of microcrack density and length. This is at odds with several previous studies, although once again, the difference in technologies used in different studies to quantitatively assess microdamage should be emphasized, namely, X-ray micro-tomography in our study versus optical–based microscopy in previous reports. Several studies report that three or four-point bending fatigue tests on calibrated cortical bone specimens induced the progression of microdamage by increasing microcrack density and length in human [13], [15] or bovine bone [12]. However there is no consensus on bone microdamage induced by *in vitro* mechanical fatigue tests. For example, a recent experiment dedicated to four-point bending test on bovine cortical bone, showed no microcrack density variation but only an increase of their average length (control: Cr.Le = 41µm±22µm/loaded: Cr.Le = 108µm±63µm) [51]. In another study on whole canine femur with comparable experimental protocol [72], a modulus loss threshold was observed, i.e. microcracks accumulation started when loading modulus loss exceeds 15%. Moreover, the average microcrack length was not significantly different between control and cyclically loaded specimens, as in the present study. It was suggested that damage initiates at tissue level as nanodamage before being visible as microcracks [72]. However, numerous studies suggest that rather than a continuum between diffuse damage and microcracks, both types of damage are different events. Diffuse damage is generally found to be mainly created in response to tensile stress [12], [13] and in young bone [73] whereas microcracking occurs preferentially under compressive stress [15] and in old bone [73].

When the microcracks leading to the specimen surface were analyzed separately, the density of short microcracks was not statistically significantly different between VOI1 (control region) and VOI2 (damage region). This is in contrast with the results obtained when we considered fully embedded microcracks only. The reason for this discrepancy remains unclear. One possibility is that pre-existing and/or preparation microcracks leading to the specimen surface release stress concentration, thus preventing significant growth of microdamage at the periphery of the specimens. On the contrary, microcracking process, as an essential mechanism inside bone specimen to release stress concentration, may explain why small microcracks density increases. Further experiments are required to elucidate this issue.

Microcracking process is well known in fiber-reinforced composite materials as the so-called three-phase modulus degradation curve [74]. The evolution of bone microdamage characteristics (density and length) throughout modulus degradation was observed for the first time in bovine tibia during tension fatigue cycling [14], [16]. O’Brien observed that the formation of new microcracks was initiated during an early phase of damage, which confirmed the above mentioned hypothesis. New microcracks grew up to reach a maximal length of about 100µm mainly due to cement lines acting as barriers [75], while only 6% of the native microcrack propagated. Thus, the level of damage depends on the phase of the modulus degradation curve. In early stages of fatigue, damage first manifests by short microcracks, confined to interstitial bone tissue, releasing and redistributing local stress in order to enhance fatigue life, as it was suggested by Sobelman [15].

Our results seem to be in line with the progressive growing of microdamage described in [14], [16]. Indeed, we observed a doubling of the shortest microcracks density Cr.Dn.Q1 (those in the first quartile) without an increase of the global microcracks density, neither an expansion of the average length of the pre-existing microcracks. It is likely that our fatigue cycling protocol was not sufficiently strong to increase the average length of the pre-existing microcracks. Nevertheless it was sufficient to lead to the formation of new short microcracks, revealed by the increase of Cr.Dn.Q1. This suggests that the final damage state of our specimens remained low compared to the previous studies [12], [13], [15], [51]

The significant correlation found between the variation of *α_f_* and the variation of Cr.Dn.Q1 is suggestive of the sensibility of the nonlinear hysteretic elastic parameter to newly formed microcracks in early phases of bone damage. However we cannot reject an effect of early diffuse or nanodamage to hysteretic nonlinearity as this damage was not investigated, neither the initiation and growing of a single macrocrack (i.e. known to produce large nonlinear elastic behavior) that was not contained by the microcracking process.

## Conclusion

Altogether our results evidence:

1.   an increased number of short microcracks in damaged   regions compared to the load-free (control) regions. These   shortest microcracks, with length in the first quartile, are   suspected to be newly formed microcracks as a result of   fatigue cycling [14];
2.   a significant effect of damage steps on the nonlinear   hysteretic elastic parameter *α_f_*.
3.  a significant relationship between the relative variation of  nonlinear elasticity and the relative variation of the density  of newly formed microcracks.

The hysteretic nonlinear parameter (*α_f_*) is sensitive to early bone microdamage. Our results suggest that NRUS can be used to monitor fatigue microdamage in *in vitro* experiments. The ability to non invasively quantitatively assess microdamage accumulation in living bones would represent an important step to improve our understanding of skeletal status. However, several scientific and technical problems have to be solved first, such as the adaptation of the nonlinear ultrasound techniques to *in vivo* measurement requirements.
